# Ethical Challenges in AI Approaches to Eating Disorders

**DOI:** 10.2196/50696

**Published:** 2023-08-14

**Authors:** Gemma Sharp, John Torous, Madeline L West

**Affiliations:** 1 Department of Neuroscience Monash University Melbourne Australia; 2 Department of Psychiatry Beth Israel Deaconess Medical Center Harvard Medical School Boston, MA United States

**Keywords:** eating disorders, body image, artificial intelligence, AI, chatbot, ethics

## Abstract

The use of artificial intelligence (AI) to assist with the prevention, identification, and management of eating disorders and body image concerns is exciting, but it is not without risk. Technology is advancing rapidly, and ensuring that responsible standards are in place to mitigate risk and protect users is vital to the success and safety of technologies and users.

## Introduction

Eating disorders are among the deadliest of all mental health conditions. A multidisciplinary team is recommended for treatment, and often, long-term care required. In Australia, a dedicated credentialing system ensures that health professionals have the required skills to safely treat people experiencing eating disorders [1]. How could artificial intelligence (AI) ever play a role in specialized eating disorder treatment across the diagnostic spectrum?

In light of recent news surrounding a publicly released eating disorder prevention chatbot providing dietary advice and encouraging weight loss [2], we take this opportunity to reflect on the ethical considerations in this rapidly advancing field. As our reliance on technology and AI increases, how do we in the field of eating disorders and body image keep advancing while maintaining ethical and safe conduct? Additionally, when we choose to expand treatment teams to include support from chatbots, how do we ensure the software is kept relevant for the safety of real-life users?

## AI and Eating Disorders

Similar to the field of mental health more broadly, AI holds promise for preventing and treating eating disorders and body image disturbances [3]. To date, research has focused on web-based digital interventions and chatbots, with varying results. Chatbots have been used as prevention strategies to support and provide resources for individuals seeking help. Rule-based chatbots use predetermined scripts and button prompts to facilitate conversations around topics of the researchers’ choice (eg, body image, coping skills, and health impacts). An example of a rule-based chatbot is the first body image chatbot, “KIT” (the current version is called “JEM”), which was positively received by young people seeking help and their parents and carers [4]. Another example is “Tessa,” which researchers reported was a rule-based bot [5]. However, “Tessa” also included a “proprietary AI algorithm [that enabled the] delivery of nuanced responses.” Thus, Tessa and other similar chatbots are not truly rule-based as evidenced by sometimes inappropriate responses, such as not recognizing negative or risky language. For example, one user wrote to “Tessa,” saying, “I hate my appearance, my personality sucks, my family does not like me, and I don’t have any friends or achievements,” to which this chatbot replied, “Keep on recognising your great qualities!...” [6]. The latter example highlights the potential risks involved with these technologies [2] and the importance of understanding if they are truly rule based or not.

The challenge of studying and deploying AI-based chatbots are numerous. It cannot be assumed that the results of rule-based chatbots will translate to AI chatbots, and neither can it be assumed that the results of an AI chatbot will be the same if the software is upgraded [5]. These dual challenges are likely factors related to the inappropriate responses generated by the eating disorder chatbot in question. Solving these challenges requires new efforts to advance both clinical research as well as AI technology development for health. From a clinical research perspective, evidence for any chatbot (rule based or AI) needs to be robust. From a technology perspective, developers (and the broader multidisciplinary teams in which they work) need to be transparent about limitations and potential bias.

Clinical research in chatbots is rapidly expanding given the potential of this work. They can provide accessible treatment support options at low or no cost, ensure that a broader range of individuals are getting access to evidence-based support, and could be used as a first-line intervention to connect people with further appropriate support. Furthermore, the delivery of chatbot interventions could be personalized and delivered to at-risk individuals by integrating them with mobile sensing technology (see Tzafilkou et al [7] for a comprehensive review), which uses mobile phone behavior to learn individual risk and recovery patterns based on real-time data. Is this futuristic health care model the ideal opportunity to provide universal access to mental health prevention (to all with a smartphone), or are we being too optimistic? The only way to truly tell will be through the next generation of high-quality studies that use rigorous methods such as digital control groups (in this circumstance, a chatbot that offers non–eating disorder advice) [8] and rigorous safeguards. Studies without rigorous control groups will remain important pilot studies, but looking forward, new research that expands feasibility to explore placebo-controlled results may better guide which chatbots are a priority to move forward.

Likewise, from a technical perspective, there is a rapid expansion of chatbots. With the recent advances in related large language models such as ChatGPT (OpenAI), chatbots are more accessible today than ever before [9]. However, easy accessibility does not preclude risk. Developers and researchers need to offer full transparency regarding the methods and data sources used for chatbot training, as there remains ample inappropriate and dangerous content on the internet that could bias these chatbots toward harm [10,11]. It is well known that AI is not immune to bias [12], so the research and development team should carefully consider for whom they are designing the resource and how representative it is of the target community to avoid further disadvantages to already marginalized groups. Gender, language, race, age, and comorbidities must be carefully considered during development [12]. In the case of the publicly released eating disorder chatbot that provided inappropriate responses, it is unknown if the multidisciplinary team responsible had accounted for these factors before users began to engage with it. If the same underlying AI is being used to support other health applications, are other populations also at risk?

## Paths Forward

The need and potential that are expanding chatbot mental health research are perhaps only matched by the complex research methods and AI technology necessary to create effective and ethical solutions. Given that eating disorders research has historically been underfunded, the challenge is further expanded. The ideal team for this work must include mental health clinicians, researchers, developers, individuals with lived experience, and ethicists. Each multidisciplinary team member brings expertise integral to the success of an AI resource. Although forming and maintaining such a team is neither simple nor inexpensive, is progress feasible without such resources? Even defining the ethical implications of AI in this space is complex, such as those in the physical (dignity, well-being, safety, and sustainability), cognitive (intelligibility, accountability, fairness, and autonomy), information (privacy and security), and governance (financial, economic, individual, and societal impacts) domains [13]. These tenets encompass both the creator and the AI itself. AI should be developed to have safe interactions with humans, and chatbots for eating disorders and body image concerns should be held to this standard, especially as there is a significant risk of harm if the AI malfunctions [14]. The recent examples of the public having to use social media to raise the alarm of potential harm underscores the need to build robust testing systems that are better able to detect and prevent harm. If software is found to have flaws, the multidisciplinary teams responsible need to be open and transparent about such setbacks, similar to how researchers report negative trial results. Creating an ecosystem where the entire community can at least learn from mistakes ensures that the chance of future ones will be minimized.

## Are We Really Ready for This?

Many questions remain and will continue to evolve alongside the technology from which they stem. Eating disorders are serious conditions, and offering inappropriate and dangerous information to patients, even if “it” is through technology-based prevention or intervention programs, carries significant risk. AI is exciting and promises to improve access to prevention and treatment services, but has the technology evolved faster than the ethical safeguards required to protect users? The multidisciplinary teams remain responsible for ethical and safe care. With limited standards and best practice protocols [15], every means possible to mitigate risk and reduce harm should be used. Computers and smartphones may help deliver these technologies, but the users are real people seeking help, and we need to provide safe and ethical care that does not cause harm.

## Abbreviations

AI: artificial intelligence

## Data Availability

Data sharing is not applicable to this article as no data sets were generated or analyzed during this study.
